# Does average volume-assured pressure support make any difference compared with BIPAP?

**DOI:** 10.1186/cc13455

**Published:** 2014-03-17

**Authors:** G Canpolat, A Ozgultekin, G Turan, A Iskender, E Adiyeke, O Ekinci

**Affiliations:** 1 Haydarpasa Numune Teaching and Research Hospital, Istanbul, Turkey

## Introduction

Average volume-assured pressure support (AVAPS) has been developed to ensure a more fixed tidal volume along with the convenience and advantages of pressure support ventilation. In this study we compared the AVAPS with the BIPAP.

## Methods

Approval was obtained from the hospital's ethics committee for the study. Thirty-three patients over 18 years of age with acute respiratory failure as a result of either internal or surgical reasons were included in the study. This study was conducted with the Philips V 60 ventilator, which includes both BIPAP and AVAPS mode. The implementation protocol for non-invasive ventilation (NIV) included, firstly, a 2-hour BIPAP application (Period Bi) and then, without interruption, a 2-hour AVAPS application (Period AV). After measuring the basal blood gas analysis, patients were informed of how NIV would be practiced and what function it would have. I n BIPAP mode, the ventilator parameters were adjusted as follows; EPAP: 5 to 7 cmH_2_O, IPAP: 15 to 20 cmH_2_O. Patient comfort was analyzed with a scale ranging from 0 to 2 (0: compatible, 1: medium-compatible, 2: noncompatible). During BIPAP ventilation, levels of arterial blood gases (pH, pO_2_, pCO_2 _and SPO_2_), comfort scale and hemodynamic data were recorded at the 30th minute, first hour and second hour. After the patient was monitored for 2 hours in BIPAP mode, the mode was changed to the AVAPS by setting the required rates without removing the mask. EPAP settings were adjusted as follows for AVAPS: 5 to 7 cmH_2_O, Pmin to max: 10 to 25 cmH_2_O, tidal volume: 6 to 8 ml/kg. As in the BIPAP mode, we analyzed and recorded the rates of arterial blood gases, comfort scale and hemodynamic parameters at the 30th minute, first hour and second hour. In case of agitation that prevents NIV, patients were sedated by dexmedetomidine.

## Results

When we analyzed patients according to their body mass indexes (BMI), pH and pCO_2 _values of the patients with bMi ≥30 showed a greater improvement at all three measurements in the AVAPS compared with BIPAP (*P *< 0.001). When patient compliance was examined, the number of patients regarded as comfortable in the BIPAP period was 20 (66.7%), but this figure was 25 (83.3%) for the AVAPS.

## Conclusion

Patient comfort was higher and need for sedation was lower in AVAPS. According to the results obtained from this study, the AVAPS had positive effects on pH, gas variation and patient comfort; therefore, it can be confidently used in clinical practice.

